# Evaluation of Animal-Based Indicators to Be Used in a Welfare Assessment Protocol for Sheep

**DOI:** 10.3389/fvets.2017.00210

**Published:** 2017-12-11

**Authors:** Susan E. Richmond, Francoise Wemelsfelder, Ina Beltran de Heredia, Roberto Ruiz, Elisabetta Canali, Cathy M. Dwyer

**Affiliations:** ^1^Animal Behaviour and Welfare, Animal and Veterinary Sciences Group, Scotland’s Rural College, Edinburgh, United Kingdom; ^2^Animal Production, Neiker-Tecnalia, Vitoria-Gasteiz, Spain; ^3^Department of Veterinary Science and Public Health, University of Milan, Milan, Italy

**Keywords:** sheep, welfare assessment, animal-based measures, behavior, health

## Abstract

Sheep are managed under a variety of different environments (continually outdoors, partially outdoors with seasonal or diurnal variation, continuously indoors) and for different purposes, which makes assessing welfare challenging. This diversity means that resource-based indicators are not particularly useful and, thus, a welfare assessment scheme for sheep, focusing on animal-based indicators, was developed. We focus specifically on ewes, as the most numerous group of sheep present on farm, although many of the indicators may also have relevance to adult male sheep. Using the Welfare Quality^®^ framework of four Principles and 12 Criteria, we considered the validity, reliability, and feasibility of 46 putative animal-based indicators derived from the literature for these criteria. Where animal-based indicators were potentially unreliably or were not considered feasible, we also considered the resource-based indicators of access to water, stocking density, and floor slipperiness. With the exception of the criteria “Absence of prolonged thirst,” we suggest at least one animal-based indicator for each welfare criterion. As a minimum, face validity was available for all indicators; however, for many, we found evidence of convergent validity and discriminant validity (e.g., lameness as measured by gait score, body condition score). The reliability of most of the physical and health measures has been tested in the field and found to be appropriate for use in welfare assessment. However, for the majority of the proposed behavioral indicators (lying synchrony, social withdrawal, postures associated with pain, vocalizations, stereotypy, vigilance, response to surprise, and human approach test), this still needs to be tested. In conclusion, the comprehensive assessment of sheep welfare through largely animal-based measures is supported by the literature through the use of indicators focusing on specific aspects of sheep biology. Further work is required for some indicators to ensure that measures are reliable when used in commercial settings.

## Introduction

The global sheep population is over 1.2 billion animals (1), bred primarily for milk, meat, and wool production. The majority of these animals are managed under extensive conditions, where at least a portion of their life is spent outdoors on grazing land with minimal daily interactions with humans (2). The public perception is that these animals live a “natural life” free of welfare constraints (3). However, extensive systems do not automatically guarantee high welfare standards and these systems often pose unique and complex problems (4). For example, extensively managed sheep face an increased risk of predation compared to housed animals, they may not have sufficient shelter from extreme weather, and may go weeks or months without inspection, such that identification or treatment of welfare problems does not occur promptly if at all (4). In assessing sheep welfare, identifying the risk of experiencing poor welfare, because the systems are infrequently monitored, is important as part of welfare assessment, as well as indicators of actual welfare compromise. For example, poor fleece coverage can be a risk factor for experiencing thermal discomfort if the weather is bad.

A number of studies have identified the main welfare problems of sheep, and a few studies have provided a welfare assessment scheme (5–7), or identified welfare indicators (8–10). However, these considered mainly housed sheep (5–7), used abattoir-based measures (10), relied heavily on resource-based indicators, and/or focused on animal health indicators (9), and did not provide a comprehensive welfare assessment scheme as was achieved for other species in Welfare Quality (11). The Animal Welfare Indicators (AWIN) project followed Welfare Quality, and developed similar welfare assessment protocols for horses and donkeys (12), goats (13), turkeys (14), and sheep. This study describes the first steps to designing the comprehensive AWIN welfare assessment protocol for sheep, using animal-based welfare indicators. Animal-based indicators, or outcome measures, are generally considered as more indicative of animal experience than input measures, or resource-based indicators and have become the preferred method of assessing welfare (e.g., Welfare Quality protocols). The protocol focuses on ewes as the main sheep type present on all sheep farms, and whose welfare might be considered to be the most reliable indicator of on-farm welfare, as adult ewes will generally remain on the same farm year round for several years. The work primarily considers indicators relevant to sheep in extensive, unhoused environments, although consideration of housed sheep is also included as many flocks will have a housed phase in the production cycle.

## Materials and Methods

The 4 principles and 12 criteria outlined in the Welfare Quality project (15) were used to develop the list of potential sheep welfare indicators to be evaluated in this project. The principle of “Good Housing” was renamed “Good Environment” to be applicable to animals in both housed and non-housed conditions (e.g., 50% of UK sheep flocks are never housed, most sheep production systems involve at least some outdoor management). The criterion “ease of movement,” however, was considered to be only applicable to housed sheep.

A list of candidate animal-based measures for each criterion was developed by performing a literature search using the online database Web of Knowledge (http://apps.webofknowledge.com/). All databases were included in the search, the timespan was set to include the earliest possible year (1864) to the present and the language filtered to English. The search terms “sheep,” “welfare,” and “indicator” were initially used. In order to capture as many potential indicators as possible additional searches were conducted using the terms “assessment”; in place of “indicator,” and “pain” in place of welfare as well as additional searches for each criteria using those and related terms (e.g., for the criteria: absence of prolonged hunger, search terms “hunger,” “undernutrition,” and “malnutrition” were used). If no suitable indicators were yielded from these searches, the terms were also widened to include other ruminant species (goats and cattle). Initial searches were conducted in 2011 and supplemented by a later search in 2016 to account for new developments in the literature. The literature obtained was cataloged based on their applicability to the four Welfare Quality principles and criteria.

The feasibility of measurement for housed and unhoused sheep (time efficient), the validity (relevance to sheep welfare), and reliability (produce consistent results when performed at different time points or by different assessors) of each putative welfare indicator was then assessed. Evidence in support of validity and type of validity available, reliability, and feasibility for on-farm assessment was gathered from the literature where available (see Tables S1 and S2 in Supplementary Material). To refine this list, and to provide face (agreement that the measure seemed relevant to the welfare issue) and consensual (agreement that the measure was valuable) validity, an email consultation of six sheep welfare experts was conducted, followed by an expert meeting during which five animal welfare and production scientists (from UK, Spain and Italy, with experience (3–20 years) in sheep welfare and production) discussed each indicator in detail. Indicators were accepted, rejected, or selected for further evaluation and development on the basis of their validity, reliability, and feasibility. Although our focus was on outdoor managed animals, many of the indicators have only been developed and tested in an indoor situation. In these cases, we also assessed whether the measure could be valuable in an outdoor environment. Where no suitable indicators for a criterion were available in the literature, or were not generated during the email consultation, the expert panel discussed other relevant animal-based measures. If no suitable indicators were then derived, resource-based indicators were considered.

Where animals are not housed, identification of individuals may be difficult and a large flight distance may prevent assessors approaching and handling the animals. Gathering for inspection may be difficult, may alter welfare state, and might be unsuitable at particular times of year, e.g., when lambs are present. Thus, consideration of whether animals would require gathering and inspection at close quarters for the indicator to be measured was also included.

## Results and Discussion

An initial list of potential sheep welfare indicators derived from the literature was developed (see Tables S1 and S2 in Supplementary Material) where, as a minimum, face validity was present. At least one putative animal-based indicator was suggested for each criterion, although a number of resource- and management-based indicators were also included. The evidence supporting or refuting the use of each indicator is outlined below for each Principle and Criteria (see also Tables S1 and S2 in Supplementary Material for summary).

### Good Feeding: Absence of Prolonged Hunger

Three potential indicators are suggested for this criterion: assessment of body condition score (BCS) by manual palpation of the lumbar spine, assessment of tooth loss, and assessment of lamb mortality from farm records.

#### Body Condition Score

Body condition score assesses the amount of fat and muscle overlying the spine: low values occur when energy expenditure exceeds intake and body fat is mobilized to meet the animal’s needs, whereas high values can indicate over-feeding or excessive confinement (3). Convergent validity for BCS has been demonstrated as BCS covaries with indicators of biological function such as health, fertility, and mortality (16) and is correlated with plasma concentrations of non-esterified fatty acids and glucose [indicators of tissue mobilization (17)]. Furthermore, thin ewes have higher feeding motivation than ewes with higher BCS (18) and are at greater risk of developing pregnancy toxemia (17).

Evidence for the reliability of BCS scoring is conflicting: some studies report low levels of reliability (19) and some extremely good agreement (7, 20). Inconsistency in the methods used may account for some of this variation as both inter- and intra-observer reliability has been found to improve when assessors used a half-point scale compared to the full-point scale (9), and following training. An alternative scale that identifies only those animals that are considered too thin or too fat has also been proposed for welfare assessment (8) as this identifies only those animals considered to be a welfare risk.

Body condition score assessment requires that animals are gathered and handled. However, the method is quick and simple and is already used on farm by many managers to monitor feed intake levels (21), thus there is good on-farm acceptability of this measure.

#### Tooth Loss

Grazing sheep rely on their lower incisors (upper incisors are absent) to bite, whereas the molars (upper and lower) grind down the cell walls of forage. Loss of the permanent incisor teeth is a major factor in culling of adult sheep (22) as incisor wear, damage and loss has been shown to affect feed intake leading to a reduction in weight gain, BCS, and milk and wool production (22, 23). Using tooth loss as a welfare indicator may allow at risk animals to be identified sooner, although housed animals may not experience a reduced intake through loss of incisors.

The reliability of assessing sheep dentition has not been tested. As with BCS, assessment of tooth loss requires handling of sheep, but assessment is quick and simple. Assessing the mouths of ewes is also a frequently conducted on-farm procedure suggesting good acceptability.

#### Lamb Mortality

Adequate maternal nutrition has been extensively demonstrated to be essential for lamb survival [e.g., Ref. (24)]. Undernourished ewes that produce lambs of low birth weight, with impaired neonatal behavior and poor ability to thermoregulate (25), show reduced expression of maternal behavior (26) and a lower availability of colostrum and milk. Overweight ewes are also at risk of metabolic disorders and increased lamb mortality. In addition, lamb productivity (lambs weaned per ewe mated) is positively correlated with overall farm welfare score (27).

Assessing lamb mortality requires adequate farm record keeping. Many farms do not keep records of lamb mortalities. However assessment of some measure of lamb productivity is possible with even rudimentary farm records (27), although these fail to distinguish between different causes of mortality. Improved record keeping would improve the reliability of this measure, as seen in other datasets [e.g., Ref. (28)]. Lamb mortality can be affected by a number of other factors, including maternal disease state, maternal stress, stocking density, and management [e.g., Ref. (29)], thus this indicator is not specific for absence of prolonged hunger. However, this lack of specificity can also mean that lamb mortality may function as an “iceberg” indicator for more than one welfare condition.

The perceived simplicity of using farm records to obtain information regarding the number of lambs weaned per ewe implies good feasibility; however, lack of even basic records may restrict feasibility in some systems. Productivity can also be influenced by breed and system, and high productivity does not necessarily indicate good welfare, thus this indicator should only be used to assess poor productivity against a background of what should be achievable with a given breed and system.

#### Conclusions: Absence of Prolonged Hunger Indicators

All three indicators meet the minimum requirements of validity, reliability, and feasibility, although reliability of tooth loss and lamb mortality requires further work. Given that tooth loss and BCS, both require handling, are simple to use and BCS is a more direct method of assessing prolonged hunger in all animals, BCS is the preferred indicator. Although lamb mortality has poor specificity, it has potential to act as an “iceberg” indicator by integrating a number of possible welfare challenges experienced by the ewe.

### Good Feeding: Absence of Prolonged Thirst

Three potential indicators were identified for this criterion. Of these, assessments of plasma constituents were discarded as impractical for welfare assessment. The two remaining possible indicators were as follows: a skin-pinch test and the resource-based assessment of water availability.

#### Skin-Pinch Test

A skin tent test (time taken for skin to lie flat following a pinch, derived from human measures of dehydration) has been used in working equids to assess dehydration. However, the convergent or construct validity of this measure has not been successfully demonstrated (30). For wool sheep, there are few sites on the animal where this test could be successfully conducted, thus feasibility of this measure is questionable.

#### Access to Water

Validity of the relationship between ready access to water and absence of prolonged thirst is implicit and no studies have explicitly examined this. Many sources of water for extensively managed sheep may be natural and whether the sheep can safely access a water course may need to be assessed. In addition, dirty or contaminated water courses, whether natural or man-made, will also reduce palatability.

Although studies do not appear to have assessed this, it is likely that reliability will be high, as is generally found for resource-based measures. Similarly, unless natural water sources are widely dispersed or hard to find, this measure can be readily determined in both indoor and outdoor managed sheep.

#### Conclusions: Absence of Prolonged Thirst

Available animal-based measures for assessing absence of prolonged thirst are not valid or feasible. Therefore, only a resource-based measure, access to water, is proposed for this criterion.

### Good Environment: Comfort around Resting

Three possible indicators were suggested from the literature for this criterion: time spent lying, lying synchrony (whether all sheep could lie down simultaneously), and coat cleanliness.

#### Lying Time

Lying time is reduced when there is less space available (31, 32), particularly in subordinate animals, and in shorn ewes when housed on solid or slatted floors compared to straw bedding (33). Rams also increase time spent lying when provided with plastic mats over wire mesh floors (34). These data suggest that sheep reduce lying time when there is insufficient comfortable resting area, thus assessing lying time reflects the ability of animals to lie in comfort.

The reliability of lying time as a measure of welfare has not been tested in sheep, although good reliability is reported in cows (35). Time spent lying increases with stage of gestation (32), decreases with re-grouping or mixing of sheep (36) or separation of ewes and lambs (37), and increases or decreases with disease [e.g., lameness (38); sheep scab infestation (39)]; therefore, this measure is not specific to the provision of a comfortable resting area.

With sufficient space in an indoor environment sheep lay for nearly 70% of an observation period (31), suggesting that assessing lying time may be feasible. However, outdoor managed animals have a pronounced circadian rhythm of active and resting periods (40), and time spent lying during daylight hours may be much lower than in housed environments. Circadian rhythmicity may mean that the timing of observations will have a marked impact on assessments of lying time, and a prescriptive period when these observations should be made would be impractical. Future developments in sensor technology may allow this measure to be recorded remotely and continuously which could lead to a re-evaluation of its utility.

#### Lying Synchrony

Groups of animals that can perform lying or feeding behavior synchronously have adequate space and access to resources without the need for competition (6). The proportion of time where sheep are able to lie simultaneously is markedly reduced with less space allowance (31), and an increase in movement and disturbance occurs at high stocking density (32). A high degree of synchrony of resting or grazing behavior within a herd or flock is considered to be indicative of a positive welfare state, particularly for subordinate animals (6).

Reliability for this measure has not been assessed. Although individual variation in lying behavior, as described above, may influence synchronicity, lying simultaneously is likely to be more specific to the availability of comfortable resting area than is individual lying time. Assessment of synchronous behavior in undisturbed animals can be quicker and more readily assessed than lying time. This measure is also less likely to be influenced by circadian changes in behavior, except while the groups are transitioning between active and inactive phases.

#### Coat Cleanliness

Coat cleanliness can provide information on whether sheep have been forced to lie in wet or muddy areas. Consensual and face validity for coat cleanliness as a sheep welfare indicator has been shown (8). However, convergent validity of fleece cleanliness and environmental conditions is lacking. Stubsjøen et al. (7) assessed coat cleanliness of housed sheep and the hygiene of the lying area, although did not report on the relationships between these measures.

The inter- and intra-observer reliability of a binary coat cleanliness scale has been shown to be high (9), and a four-point scale based on the Animal Needs Index scale was also found to have good inter-observer reliability when applied to housed sheep (5). Coat cleanliness may be influenced by immediate environmental conditions when animals are handled (e.g., cleanliness of handling pens), but is more specific to the conditions in which the sheep live when animals are not first gathered before assessment. As this measure does not require the animals to be gathered and handled, it is feasible for this measure to be performed simply in undisturbed animals in their home environment.

#### Conclusions: Comfort around Resting

Lying synchronicity and coat cleanliness show the most promise for further use in welfare assessment. Lying time is likely to be a difficult measure to apply in the field at present, and assessment of the ability of sheep to lie simultaneously may provide sufficient information more simply. Validation of coat cleanliness as a measure beyond the consensual and face validation so far available would be beneficial.

### Good Environment: Thermal Comfort

Three measures for assessing thermal comfort that could be practically possible to implement were suggested: increased respiration rate and panting, shivering, and measurement of rectal temperature. The resource-based measure of access to shade or shelter was also considered.

#### Increased Respiration Rate and Panting

In wooly sheep, dissipation of heat through sweating is severely reduced, so sheep rely on behavioral mechanisms (seeking shelter) and heat loss from the respiratory tract (41). The initial respiratory response is an escalation of breathing rate, followed by slower heavy panting with the mouth open and tongue protruded (42). A respiration rate above 40 breaths per minute is considered to be indicative of panting (41) and increased respiration rate is reliably associated with increasing environmental temperature (43).

The reliability of using panting as an indicator of heat stress was attempted by Phythian et al. (9), however the incidence of panting was too low (in outdoor managed sheep in the UK) for analysis. Assessment of reliability under conditions where heat stress may be more prevalent is required. Panting may also occur in sheep under psychological stress, when stress-induced hyperthermia can occur (44), thus this measure is specific for heat stress only when measured in undisturbed animals, but can be an indicator of distress under other conditions.

#### Shivering

Shivering is the main mechanism used by adult sheep to generate heat. However, sheep are very resistant to cold and their lower critical temperature can be less than 0°C in fully fleeced adult sheep (45), thus shivering may only be infrequently observed in adults.

The reliability of visible shivering does not appear to have been assessed for sheep, either because it occurs at too low an incidence to be assessed or because the presence of the fleece makes observation difficult, suggesting that this is not a feasible measure in sheep.

#### Rectal Temperature

Direct measurement of temperature can clearly provide a useful assessment of body temperature. However, sheep are efficient thermoregulators and can maintain core body temperature for several hours, even in extremes of temperature (45), thus rectal temperature may not accurately reflect the effort involved in maintaining thermal homeostasis. This measure also requires animal handling and stress-induced hyperthermia may influence the validity of the results. The invasive nature of this measure may compromise biosecurity and it is unlikely to be acceptable for on-farm welfare assessment.

#### Access to Shade and Shelter

Sheep use behavioral mechanisms, such as seeking shelter or shade, as part of their ability to adapt to thermal extremes. Sheep are able to maintain body temperatures even at high ambient temperature with provision of shade, but unshaded sheep had higher respiration rates, higher plasma cortisol and lower indicators of mobilization of body fat than sheep with shade at high ambient temperatures (46, 47). Adult sheep in full fleece seek shelter only when they are outside their thermoneutral zone, which can occur infrequently in temperate sheep (48). However, shorn sheep, and those with thin fleeces, do make more use of shelter, particularly on windy days. In addition, provision of shelter can have a significant impact on improving lamb survival (49).

The use of access to shade and shelter does not appear to have been used before in welfare assessment, thus its reliability is untested. However, it is a feasible indicator to assess on farm.

#### Conclusions: Thermal Comfort

Panting, and elevated respiration rate, is an important and useful indicator of heat stress which is likely to be very relevant for sheep in hot environments, and housed sheep in full fleece. No animal-based measures of cold stress were considered acceptable, thus this aspect of welfare may best be measured by the resource-based measure of access to shelter.

### Good Environment: Ease of Movement

This criterion is only of relevance to housed ewes, where two suggested that animal-based indicators were considered: aggression and displacements, and hoof overgrowth. The resource-based indicators, stocking density and floor slipperiness, were also considered.

#### Stocking Density

Reduced space through increased stocking density is associated with decreased activity and lying time (31, 32), a decreased immune response to challenge (3) and increased fecal glucocorticoid metabolites (50) compared to lower stocking densities.

The speed and ease of calculation of space availability per animal makes this assessment feasible for an on-farm welfare assessment, and this measure has been used in other farm studies [e.g., Ref. (5)].

#### Floor Slipperiness

This has face validity with ease of movement and has been used in welfare assessment for sheep (5) but no studies have associated perceived slipperiness of flooring with incidence of slips, falls, and difficulty in movement. In tests with two observers, floor slipperiness, as a component of wider environmental assessment, was found to have high reliability (5).

#### Aggression and Displacements

In housed sheep, lying space is an important resource and competition can lead to aggression and social stress (33). This is exacerbated when space is restricted, increasing the frequency of displacements (31, 51). Low space allowance is also associated with an increased frequency of both positive and negative social contacts (32).

By combining the agonistic and displacement behaviors of cattle, good inter and intra-observer reliability has been found (52). Further work is required in order to determine whether this is true for housed sheep. Aggression and displacement also occurs with other forms of competition, such as access to feeders, so is not specific to lying space.

#### Hoof Overgrowth

The hoof is worn by sheep when walking on hard or rocky surfaces. A small space allowance reduces walking time in sheep (3, 32), reducing wear on the horn, although no studies have directly linked reduced movement with an increase in hoof overgrowth. This measure will also be influenced by the frequency with which hooves are trimmed as a management action.

Hoof overgrowth has been measured in sheep welfare assessment (5) and inter-observer reliability found to be very good. Claw overgrowth may, therefore, be a potential indicator of ease of movement in housed sheep. However, hoof wear can be affected by lameness, which prevents the animal from eroding the hoof, thus an elongated hoof may indicate lameness rather than an inability to move easily in a housed environment. The prevalence of lameness and claw overgrowth is known to increase in housed animals in comparison with outdoor grazing (53).

#### Conclusions: Ease of Movement

Both animal-based and resource-based measures have some applicability to the assessment of ease of movement. Of the animal-based measures, the assessment of aggression and displacements currently has greater validity, although its reliability still requires testing. Stocking density is also straightforward to measure and has consistently been shown to be associated with reduced welfare in housed sheep.

### Good Health: Absence of Injury

This criterion is assessed by a single indicator assessing the degree of integument alteration present. Injuries that might cause altered gait are considered under Good Health: Absence of Disease (4.7), internal injuries are considered under general behavioral responses indicative of pain (4.8).

#### Integument Alteration

Validity of this measure is assumed since it is a direct assessment of the presence of injury involving cuts and wounds. The reliability of assessments of skin lesions and wounds has been calculated by a number of authors and is suggested to be very good (3, 5, 7, 9). Assessing integument alteration requires handling in sheep as presence of a wooly coat will obscure most injuries to the body. However, this assessment can be readily conducted in handled animals.

### Good Health: Absence of Disease

The most common endemic diseases of sheep are lameness, endo- and ectoparasites, eye disease, respiratory disease, and mastitis (8), thus the indicators to assess this criterion reflect this prevalence.

#### Lameness (Gait Abnormality)

Lameness is generally assessed by gait scoring, with a number of possible scoring systems suggested [e.g., Ref. (54–56)]. Gait scoring is associated with the presence of and severity of foot rot lesions (the main cause of lameness in sheep) in several studies [e.g., Ref. (57, 58)]. In addition, treatment of lame sheep for foot rot reduces or eliminates lameness as assessed by gait score (59), suggesting that gait alterations are largely caused by disease. Lameness in sheep is associated with increased plasma cortisol, adrenaline, and noradrenaline (60, 61), reduced weight gain and reduced milk yield in dairy sheep (62).

Lameness can be assessed in unhandled animals and in gathered flocks. The more fine-grained assessments require the animal to walk on a hard, flat surface (56), which may not be available on all farms. However, simpler systems have been used on commercial farms with acceptable reliability [e.g., Ref. (55)], suggesting that this measure can be easily applied on a diversity of farms. The inter- and intra-observer reliability of gait scoring has good reliability (54–56).

#### Breech Soiling (Dag Score)

Fecal soiling, or dags, occurs when fecal matter adheres to the wool around the tail and legs (63). This is associated with higher gastrointestinal parasite burdens, such as fluke and nematodes leading to diarrhea (64), infrequent use of anthelmintic drugs, lower fecal consistency, poorer or wetter pasture, and lower live weights (65). The presence of fecal matter on the fleece also increases the risk of fly strike (63, 65).

Assessing dag score on farm is feasible (9, 65), and measures can be made simply on unhandled animals. Inter-observer agreement for the assessment of breech cleanliness is high (9). Fecal soiling may occur when animals are exposed to high-quality spring grass, thus this measure may not be highly specific for gastrointestinal worm burdens. However, as fecal soiling is a risk factor for fly strike, this measure remains relevant for sheep welfare.

#### Fecal Egg Count

Assessment of the presence of parasite eggs in the feces of individuals or groups assumes that there is a relationship between eggs shed and the total amount of eggs in the gastrointestinal tract (66). Different methods of estimation of fecal egg counts exist and yield results with differing sensitivities (67). However, all methods assess high or low egg counts, and individual worm species can be distinguished. Although sample collection, particularly on a group basis, can be obtained relatively simply, the method for determination of egg counts can be time consuming, requires specialist training and off-farm assessments which makes this less suitable for on-farm welfare assessment.

#### Wool and Skin Condition/Irritation

Sheep may become infested with a range of ectoparasites (mites, lice, fly larvae), which lead to itching, rubbing, biting, and depressed wool growth (68), and can be readily observed on inspection of the wool and skin. Some infestations can also lead to breaks in the wool fibers and can be seen at a distance where ewes have partially shed fleeces.

Assessment of fleece and skin condition has formed part of on-farm assessments (7, 9, 69) and can be conducted in unhandled animals (wool loss), although a thorough inspection of skin irritation requires animal handling. Good inter- and intra-observer reliability has been found for this indicator. Assessment of wool loss has high intra-observer reliability but needs additional work to assess inter-observer reliability when assessed at a group level (9). Impaired wool growth, reduced staple-strength, and increased fiber-shedding are also associated with lameness and elevated plasma cortisol (70, 71). Wool loss may not, therefore, be specific for the presence of ectoparasites, nonetheless it is potentially a useful indicator of sheep welfare affected by several welfare conditions.

#### Mucosa Color

Mucosa color has been widely assessed on a standardized color chart [FAMACHA© (72)], where it has been shown to have good correlation with the presence of *Haemonchus contortus* in sheep (64, 73). More recently, the scale has also been shown to be positively associated with the presence of another blood-feeding parasite, liver fluke (74).

The scale can be readily applied in handled animals and has been used widely in on-farm assessments of requirements for anthelmintic treatments (64). Inter-observer agreement and test–retest evaluation of the scale has found moderate reliability (75), although differences between breeds in scores with the same levels of parasitic infection rate are reported (76).

#### Eye Condition

The presence of swellings, discharge, infection, or other eye abnormalities, such as entropion, has been suggested for sheep welfare assessment (7, 69). Eye condition has formed part of welfare assessment for young lambs (55), where it has face and consensual validity (8), and can be assessed in handled animals. In lambs reliability of assessment of eye condition was considered to be good (55), although no data for ewes are available.

#### Respiratory Condition

This section will consider together the indicators of hampered respiration, nasal discharge, and coughing as there are few papers in the literature, and not all distinguish the different conditions. Respiratory infections are associated with coughing, sneezing, nasal discharge, and/or audible breath sounds (69). The frequency of each is influenced by the type of infection and the environment in which sheep are kept. However, for welfare assessment purposes, the presence of any of the symptoms is evidence of impaired respiration, due to either infectious disease or poor ventilation.

Respiratory condition can be assessed on farm in handled animals, although coughing may be more readily assessed in unhandled animals as a group measure. Binary presence/absence scales are most commonly used to assess coughing and nasal discharge (7, 9), although in these studies the incidence rates were too low to conduct reliability analyses.

#### Swollen Joints and Callus

Swellings on the knees and hocks are relatively common in dairy cattle and associated with lameness, slipping, and falling and aspects of housing design [e.g., Ref. (77)]. In housed sheep, the presence of calluses has been reported although at low incidence (7) and whether this is related to lameness or aspects of housing design has not been tested. Reliability of this indicator was found to be poor (7), although this may be related to the low incidence.

#### Udder Traits

Acute clinical mastitis is usually determined by bacteriological tests and somatic cell counts in milk accompanied by changes in the udder and other signs of ill-health such as elevated temperature (78). Assessment of somatic cell counts in dairy sheep may be possible on an individual or group basis but is not feasible for routine welfare assessment of meat sheep. Subclinical and chronic mastitis may be difficult to detect, although physical indicators such as abnormalities in skin color, shape, consistency, hardness, and presence of lesions on the udders are indicative of the condition. Teat injuries and consistency of the udder as determined by palpation have been shown to be related to the incidence of mastitis confirmed by bacteriological tests (79).

These assessments are most readily performed in dairy sheep, where udders are frequently handled, but performing a clinical assessment on animals which have been gathered may also be feasible for an on-farm welfare assessment of meat sheep when lactating (69). There are no reports of reliability assessment of scoring udder traits for welfare assessment.

#### Conclusions: Absence of Disease

The validity and feasibility of scoring lameness, breech soiling or dags, wool loss and skin irritation, mucosa color, eye and respiratory condition, and some measures of the udder to determine the absence of disease is supported by the literature. The reliability of assessing eye condition, respiratory condition, and udder traits is unknown and requires further work.

### Good Health: Absence of Pain Induced by Management Procedures

The two most common pain-inducing management procedures that ewes will undergo are those associated with identification (placing ear tags, notching, or cutting the ears) and tail docking. Both procedures are permitted, without the use of anesthetics or analgesics, in many countries, and ear tagging is mandatory in the EU. Thus, the indicators selected focus on compliance with the law and the skill with which the procedures are applied. In addition, the possibility of assessing the presence of pain in general was considered through the animal-based indicators of tooth grinding, social withdrawal, facial expression, and postures associated with pain.

#### Ear Damage Associated With Identification

Ear tag type and position affects the severity of lesions caused and the likelihood that the tag would be lost (80). On-farm assessments report that 8% of ewes have ear tags torn out (7). The reliability of assessing the presence of ear lesions, tears, notches, and missing tags or other signs of ear damage has not been formally tested. Although some forms of ear damage may occur for reasons other than as a result of management procedures, e.g., tears or cuts from environmental features, this appears to be the most likely and frequent cause.

#### Tail Docking

Tail docking has been shown to cause an increase in active pain behaviors, plasma cortisol, and pain postures (81) associated with acute pain. There is some evidence that this early exposure to pain may also have longer lasting impacts on the behavioral responses and pain perception of adult ewes (82). Some countries permit tail docking but restrict the methods, timing and length to which the tail can be shortened. Very short tail docking (where the tail does not cover the vulva of the ewe) has been associated with higher rates of carcinoma of the vulva in ewes and rectal prolapse in lambs (83). Assessment of this indicator includes whether tail docking has been carried out (which indicates previous exposure to pain) and tail length (which reflects an increased risk of other welfare challenges such as prolapse).

#### Teeth Grinding

Teeth grinding increases in frequency with experimental induction of visceral pain, alongside increases in plasma cortisol, heart rate, hyperventilation, and other clinical and behavioral signs of pain (84), and is seen in painful disease conditions [e.g., ruminal acidosis (85)]. The frequency of tooth grinding does not appear to have been included in on-farm welfare assessments before, thus reliability has not been tested. It may be feasible to make a group-level assessment of tooth grinding but individual responses are unlikely to be feasible.

#### Social Withdrawal

As a social animal sheep are highly motivated to remain within the social group. However, animals in chronic pain can display apathy, depression, and “learned helplessness” (86), seen as withdrawal from the social group. There are no reports where this assessment has formed part of an on-farm welfare assessment scheme for sheep. However, a similar measure has been included in the welfare assessment scheme for goats (“oblivion”) where appropriate reliability was reached (13). This indicator is relevant to both extensively managed and indoor managed sheep but the reliability and feasibility of its assessment has not yet been determined, particularly in a very extensive setting.

#### Facial Expression Associated With Pain

Changes in facial expression associated with pain have been reported in many species, including in sheep (87, 88). In adult ewes, facial expressions associated with pain have been seen in sheep with foot rot and mastitis, and to decline with treatment and resolution of the condition (87). Good reliability between observers is also reported. Assessing facial expression in extensive conditions is likely to be problematic, but this may be feasible in intensive management system where it requires on-farm testing.

#### Pain Postures

Assessment of abnormal standing and lying positions has frequently been used in lambs (up to 6 months of age) to assess responses to imposed painful treatment [e.g., castration, tail docking, mulesing (81, 89)]. Posture in adult ewes following abdominal surgery has also been assessed (90) and an increase in “neck twist” events with surgery reported, although similar frequencies were observed in ewes that were treated with analgesics as in those that received placebo. Thus, although postures are likely also to be related to pain in adult sheep, the data to support this are not currently available. In lambs, postures (hunched and “tucked up”) have been reliably assessed on farm (55) but have not been assessed in adult ewes.

#### Conclusions: Absence of Pain

All indicators identified as associated with the experience of pain in adult ewes have some validity, and most are feasible to measure on farm, although teeth grinding may be difficult to assess on an individual basis. Most measures, except pain facial expression and possibly social withdrawal, have not been tested for reliability, thus decisions on which are the most appropriate measures await further work.

### Appropriate Behavior: Expression of Social Behaviors

Three indicators were suggested for the assessment of this criterion: social withdrawal, vocalization, and behavioral synchrony. As social withdrawal and behavioral synchrony are not specific to this criterion, and have been discussed above, this section will only consider vocalizations.

#### Vocalizations

Vocalizations in farm animals are generally considered as an indicator of negative feelings and an increase in vocalization has been shown to be a valid indicator of poor welfare in slaughterhouses (91). Increased vocalization may also be an indication of increased fear in sheep (92, 93). Cockram (43) concluded that vocalization, specifically high-pitched bleats, was a useful measure of distress in sheep, seen with social isolation, separation from specific individuals and on exposure to novelty, although vocalization in sheep can be inhibited in the presence of predators (94). No studies have addressed the reliability of assessing vocal behavior in sheep as an on-farm welfare indicator.

#### Conclusions: Social Behavior

Social withdrawal, behavioral synchrony and vocalization frequency all have some validity as a means of assessing social behavior in sheep. None of these measures have been rigorously tested for reliability on farm, and feasibility is inferred rather than tested.

### Appropriate Behavior: Expression of Other Behaviors

In other assessment protocols (e.g., Welfare Quality^®^ protocols, 2009), this criterion assesses the ability of the animal to perform desired behaviors despite the degree of behavioral restriction or confinement to which it is exposed. For sheep, where confinement may not be as frequent as in other farmed species, we considered the ability of the environment to provide for sheep needs, as well as assessing levels of general fearfulness. Thus, the animal-based indicators suggested for this criterion are as follows: abnormal behaviors and stereotypy (housed sheep only), vigilance, and responses to surprise and novelty.

#### Abnormal Behaviors

Stereotypy in sheep is infrequent, but the performance of oral (repetitive licking, chewing, and mouthing pen fixtures) and locomotor (rearing, butting, route-tracing, weaving) stereotypies have been reported in confined sheep [e.g., Ref. (51, 95)]. Sheep also show wool-pulling or biting when housed, particularly at high stocking density and when fed a diet with low roughage (96). Stereotypy does not appear to have been assessed on farm before as part of sheep welfare assessment, thus no reliability data are available.

#### Vigilance

In wild sheep, vigilance (the “head-up” posture) is increased in environments and situations where there is greater perceived risk (97). In domestic sheep, environments lacking in complexity are associated with an increase in alarm behaviors, compared to hilly areas with more features (98). Presence of stressors in the environment also cause increased vigilance and reduced social cohesion and grazing behavior (99). Increased vigilance is also associated with pharmacologically induced anxiety in sheep (100). No data are currently available in the literature to assess the reliability of this measure for on-farm welfare assessment.

#### Response to Surprising Events

Good correlations have been found between sheep responses to surprise and reactions to other fear inducing stimuli (101). Although there have been relatively few studies, associations between unpredictable or surprising events and physiological parameters (such as heart rate) support the validity of this measure (92). The main anti-predator response of sheep is flight to a safe distance or to cover. The time taken by sheep to resume normal behavior following flight is influenced by their perception of the degree of threat (97). Thus, both the response to a surprising event, and the time taken to resume previous behavior, can form potential indicators to assess underlying fearfulness.

Welfare Quality rejected the use of a surprise test (a sudden blow of air) in their on-farm welfare assessments due to lack of feasibility (Welfare Quality^®^, cattle, 2009). There do not appear to have been any previous studies assessing the feasibility of surprising extensively managed animals with a visual startle test and, therefore, further assessment is required.

#### Novel Object Test

The novel object test is similar to the “surprise” test except that it is the reaction on exposure to the object rather than the manner in which it is presented which is tested. Forkman et al. (102) reviewed the use of novel object tests in sheep and concluded that these responses correlate with other putatively fear-evoking stimuli.

Due to the heterogeneity of sheep farms, being able to provide a standardized environment in which to conduct this test, and defining a novel object to which all sheep will not have been previously exposed, make it unlikely that this can be conducted successfully on all sheep farms.

#### Conclusions: Other Behaviors

Assessments of levels of stereotypy, vigilance, and response to surprise have some convergent validity and the potential to be feasibly measured on farm. Although the novel object test has some validity as a measure of general fear, it is unlikely to be feasible to conduct a standardized test on all farms. The reliability of all measures requires further work.

### Appropriate Behavior: Good Human–Animal Relationship

The fear response of animals toward humans relates to an absence of habituation to human contact, as may occur in extensively managed animals, or a learned negative association acquired through poor handling (103, 104). Thus, assessment of this criterion may require different methods or different values placed on the same measure when assessing extensively managed or housed sheep. The possible indicators suggested for this criterion are: human approach test, fear test (housed sheep), and response to milking (dairy sheep only). These also correspond to the three main types of response to human tests: response to a moving human, response to a stationary human, and response to handling/restraint (105).

#### Human Approach Test

This test is designed to elicit a flight response and assessing the distance to which an animal will allow a human to approach is considered a good indicator of their comfort around humans (104). Sheep flight distances are modified by animal experience, the nature of the approaching human (e.g., whether accompanied by a dog), and perceived risk (97). However, studies assessing the discriminant validity of these tests with sheep are lacking. In addition, whether the approaching human should be familiar (which may be more relevant in terms of the welfare of sheep when handled on a day to day basis) or unfamiliar (which can be better standardized across farms) is unclear.

The repeatability of individual flight distance is much lower when measured in a group than when animals are individually tested (83). Testing animals in a group remains one of the biggest problems in this area, under farm conditions where animals are generally reared in groups, especially on large commercial units (104). The overall lack of consistency and standardization between studies using these tests on farms has led to criticism and claims that it should not be used during an on-farm welfare assessment (106). Others disagree, however, and feel it offers valuable information when performed consistently (104, 105, 107).

#### Fear Test

This test assesses the reactivity of animals to a stationary human by measuring their willingness to feed in the presence of the human (108). The most calm and confident sheep were reported to be comfortable to eat, whereas the most reactive animals did not feed at all, even following feed deprivation.

This test is specific to housed animals but has been used in on-farm assessment with sheep, where it was modified such that the human was moving along a feed bunker rather than remaining stationary (7). Assessments of the repeatability of this measure are generally good and significant (108). However, the inter-observer reliability of the assessments has not been assessed.

#### Response to Milking

Reactivity to milking has been assessed in dairy animals (109, 110). There is some evidence that these reactive responses are reduced in positively handled animals compared to negative and that there is good correlation with milk cortisol (105).

Although potentially feasible and simple to measure on dairy farms, there is likely to be considerable between farm variation in parlor design, normal milking practices, and previous experience which can influence the results. Therefore, Waiblinger et al. (105) advocate using a specific test for assessing the human–animal relationship rather than assessing reactivity during a specific procedure.

#### Conclusions: Good Human–Animal Relationship

Both the human approach test and the fear test, conducted with indoor managed ewes, have the potential to be applied on farm in welfare assessment. However, both need further work to develop the details of the methods and to assess reliability of testing.

### Appropriate Behavior: Positive Emotional State

Indicators of positive emotional state are under-researched in sheep, and there is no evidence in the literature for indicators, such as specific vocalizations, facial expressions, or postures reliably associated with positive emotions. Two potential indicators were considered for this: qualitative behavioral assessment (QBA) and the expression of play behavior.

#### Qualitative Behavioral Assessment

Unlike quantitative approaches that describe which behaviors are performed by animals, QBA asks how behaviors are performed. With QBA information about body language and the way the animal interacts with the environment is assimilated and translated into qualitative descriptors such as “calm” or “agitated” (111). Convergent validity has been demonstrated with good associations found between QBA, physiology, and behavior (112, 113).

Qualitative behavioral assessment can be applied to unhandled animals and is not sensitive to environment (114), thus making it suitable for both housed and outdoor sheep populations. On-farm QBA assessments has been shown to give good observer agreement many species e.g., cattle, goats, and donkeys (115–117), although other studies suggest poorer agreement (118). A study evaluating the inter-observer reliability of observers viewing sheep video clips reported good agreement (55); however, the assessment of the inter-observer reliability and repeatability of a fixed list of terms applied to sheep on farm requires further investigation. As the fixed lists for a species QBA can contain some 20 terms, involving both positive (e.g., calm, content) and negative terms (e.g., agitated, frustrated), QBA is not considered specific only to positive emotional states.

#### Play Behavior

Play behavior has been suggested to be an indicator of positive emotional state (119) and lambs have been shown to demonstrate behaviors indicative of anticipation prior to being given the opportunity to play (120). Play in calves decreases with reduced nutrition, reduced social contact, and pain (121–123), suggesting that it is sensitive to negative emotional states.

Play in sheep appears to be largely restricted to young animals and is seen infrequently in adult animals. As the occurrence is so rare, it is likely that play will not be readily observed on farm and, thus, its utility as a welfare indicator in adult animals is doubtful.

#### Conclusions: Positive Emotional State

The most promising indicator for assessing positive emotional state in sheep is QBA as it is both valid and feasible. Further assessments of reliability in the field are still required for this indicator.

## Conclusion

From the literature, we were able to identify potential animal-based welfare indicators for all 12 welfare criteria with the exception of “absence of thirst” which can only, currently, be assessed by resource-based measures. For some indicators, the measures could only be applied in specific environments (e.g., housed animals), whereas others were common to sheep under all conditions. Convergent or construct validity was available for most indicators, and at least face validity for all except skin-pinch test, floor slipperiness, hoof overgrowth, and udder symmetry. Some indicators (skin-pinch test, lying time, shivering, fecal egg count, teeth grinding, novel object test, response to milking, play behavior) were discounted on feasibility grounds either because they could not be recorded on farm or because variation between farms would prevent standardization. For four further measures: vocalization, assessment of stereotypy, response to surprising event, and human approach test, feasibility is still required to be assessed in an “on-farm” situation. The reliability of physical and health indicators has been reasonably well established; however, for many of the behavioral indicators, this still needs to be assessed. Therefore, a comprehensive list of animal-based indicators, addressing each area of welfare concern, has been developed, which can now be tested on farm to provide additional information on reliability, feasibility, and potential redundancy between measures.

## Author Contributions

EC, RR, CD, FW, and IH were responsible for the design and conception of the work; SR, CD, IH, and RR were responsible for acquisition and collation of the materials; and SR and CD drafted the manuscript. All authors contributed to interpretation of the materials and provided comments on manuscript drafts.

## Conflict of Interest Statement

The authors declare that the research was conducted in the absence of any commercial or financial relationships that could be construed as a potential conflict of interest.
