# Supplementary material for: Evaluation of Animal-Based Indicators to Be Used in a Welfare Assessment Protocol for Sheep
Source: Front Vet Sci. 2017 Dec 11;4:210. doi: 10.3389/fvets.2017.00210 (PMC5732139; doi:10.3389/fvets.2017.00210)
Supplement: Supplementary file 1 [file Table_1.PDF]

Supplementary Material

Table 1. List of putative welfare indicators of relevance to the assessment of sheep welfare derived from the literature and expert assessment. Indicators are animal based unless otherwise stated, applicability refers to the categories of animals to which these indicators are restricted.

Welfare Principle[1]	Welfare Criteria[1]	Indicator	Applicability	Source
Good Feeding	Absence of prolonged hunger	Body Condition Score	Handled animals	Russell et al., 1969; Caldiera et al., 2007; Pines et al., 2007; Morgan-Davies et al., 2008; Stubsjoen et al., 2011
		Tooth loss	Handled animals	McGregor, 2011
		Lamb survival[3]	Farm records	Pines et al., 2007; Veksler et al., 2008; Stott et al., 2012
	Absence of prolonged thirst	Skin pinch test[4]	Handled animals	
		Plasma/urine sample, plasma proteins etc.	Handled animals	Lowe et al., 2002; Pines et al., 2007; Tadich et al., 2008
		Access to water[4]	Resource-based	
Good Environment[2]	Comfort around resting	Lying time	Undisturbed animals	Bøe et al., 2006
		Lying synchrony	Undisturbed animals	Bøe et al., 2006; Pines et al., 2007;
		Coat cleanliness	Handled and Undisturbed animals	Napolitano et al., 2008; Caroprese et al., 2009; Stubsjoen et al., 2011
	Thermal comfort	Respiration rate/panting	Undisturbed animals	Lowe et al., 2002; Sevi et al., 2002;

				Pines et al., 2007; Lovatt, 2010; Phythian et al., 2012
		Shivering	Undisturbed animals	Phythian et al., 2011
		Rectal temperature	Handled animals	Lowe et al., 2002; Sevi et al., 2002; Lovatt, 2010
		Blood or urine measures of haematocrit, plasma protein etc.	Handled animals	Lowe et al., 2002; Pines et al., 2007; Tadich et al., 2008
		Access to shade and shelter	Resource-based	Lin et al., 2012; Caroprese et al., 2012; Pollard 2006
	Ease of movement	Stocking density	Housed animals only, Resource-based	Pines et al., 2007; Caroprese et al., 2009; Averos et al., 2014
		Floor slipperiness	Housed animals only; Resource-based	Napolitano et al., 2009
		Aggression and displacements	Housed animals only	Faerevik et al 2005; Lauber et al 2012
		Hoof overgrowth	Housed animals only	Caroprese et al., 2009; Napolitano et al., 2009
Good Health	Absence of injuries	Integument alterations	Handled animals	Caroprese et al., 2009; Napolitano et al., 2009; Lovatt, 2010; Stubsjoen et al., 2011;
	Absence of disease	Lameness (gait score)	Handled and undisturbed	Caroprese et al., 2009; Napolitano et

			animals	al., 2009; Kaler et al., 2009; 2011; Stubsjoen et al., 2011; Phythian et al., 2012; 2013
		Faecal soiling of breech area (dag score)	Handled and undisturbed animals	Caroprese et al., 2009; Lovatt, 2010; Stubsjoen et al., 2011; Phythian et al., 2012
		Faecal egg count	Handled animals	Caroprese et al., 2009
		Wool and skin condition/irritation	Handled animals	Caroprese et al., 2009; Napolitano et al., 2009; Lovatt, 2010; Stubsjoen et al., 2011; Phythian et al., 2012
		Mucosa colour	Handled animals	Bath & van Wyk, 2009
		Eye conditions	Handled animals	Lovatt, 2010; Stubsjoen et al., 2011
		Eye discharge	Handled animals	Lovatt, 2010
		Hampered respiration	Handled animals	Lovatt, 2010;
		Coughing	Handled animals	Stubsjoen et al., 2011; Phythian et al., 2012
		Nasal discharge	Handled animals	Lovatt, 2010; Stubsjoen et al., 2011
		Swollen joints or callus	Handled animals	Lovatt, 2010; Stubsjoen et al., 2011
		Udder symmetry	Handled animals	Lovatt, 2010
		Udder lesions	Handled animals	Lovatt, 2010

		Udder temperature	Handled animals	Lovatt, 2010
		Presence of udder fibroids	Handled animals	Lovatt, 2010
		Milk somatic cell count	Dairy sheep only	Caroprese et al.,2009; Lovatt, 2010; Fragkou et al., 2014
	Absence of pain induced by managemen t procedures	Ear damage caused by identification procedures (notches, tears etc.)	Handled animals	Stubsjoen et al., 2011
		Tail docking – absence of full tail	Undisturbed animals	Napolitano et al., 2009
		Teeth grinding (non-specific pain)	Handled animals	Braun et al., 1992; Kania et al., 2006
		Social withdrawal	Undisturbed animals	Phythian et al., 2011
		Pain facial expression	Undisturbed animals	McLennan et al., 2016
		Pain postures (abnormal, hunched, trembling)	Undisturbed animals	Hughan et al., 2001; Kania et al., 2006; Colditz et al., 2010; Edwards et al., 2011
Appropriate Behaviour	Expression of social behaviours	Social withdrawal	Undisturbed animals	Phythian et al., 2011
		Vocalisations	Undisturbed animals	Boissy and Dumont, 2002; Cockram, 2004; Da Costa et al., 2004; Pedernera-Romano et al., 2011
		Behavioural synchrony	Undisturbed animals	Dwyer, 2004
	Expression of other behaviours	Abnormal behaviour	Undisturbed animals	Dwyer and Bornett, 2004
		Vigilance	Undisturbed animals	Boissy and Dumont,

				2002; Dwyer, 2004; Lee et al., 2016
		Response to surprise	Undisturbed animals	Dwyer, 2004
		Novel object test	Handled animals	Forkman et al., 2007; Pedernera-Romano et al., 2011; Destrez et al., 2013
	Good human animal relationship	Human approach test	Undisturbed animals	Hutson, 1982; Waiblinger et al., 2006
		Fear test	Housed animals	Lankin, 1997
		Response to milking	Dairy sheep only	Lyons, 1989
	Positive emotional state	Qualitative Behavioural Assessment (QBA)	Undisturbed animals	Wemelsfelder and Farish, 2004
		Play behaviour	Undisturbed animals	Dwyer, 2004

[1]After Welfare Quality (Keeling et al., 2008)
[2]Adaptation of original Welfare Quality criteria to meet extensively managed animals.
[3]Lamb survival is not considered to be specific for this criterion.
[4]These indicators were not present in the literature but suggested by the authors on the basis of their knowledge of sheep/other species.

Table 2. List of putative indicators of sheep welfare, summarising the evidence associated with validity, reliability and feasibility

Indicator	Validity	Reliability	Specificity	Feasibility	Comments
Body Condition Score	Yes	Inter-observer good; intra-observer not tested	Moderate	Yes, requires handling	Undernutrition frequently cited as one of the most important welfare issues for extensively manage sheep
Tooth loss	Yes	Not tested	Good	Yes, requires handling	
Lamb mortality	Yes	Relies of farm records	Not specific	Yes	Potential ice-berg indicator
Skin pinch test	Not tested in sheep	Not tested	Unknown	No	
Access to water	Yes	Not tested	Moderate	Yes	
Lying time	Yes	Not tested	Not specific	Moderate	Feasible only in housed animals or small paddocks; may require long observation periods
Lying synchrony	Yes	Not tested	Not specific	Yes	
Coat cleanliness	Yes	Very good	Good	Yes	
Respiration rate/panting	Yes	Not tested	Good	Yes	
Shivering	Yes	Not tested	Good	No	Very low rate of occurrence in adult sheep
Rectal temperature	Moderate	Not tested	Moderate	No	
Access to shade and shelter	Yes	Not tested	Good	Yes	
Stocking density	Yes	Not tested	Good	Yes	Only suitable for housed

					animals
Floor slipperiness	Not tested	Very good	Moderate	Yes	Only suitable for housed animals
Aggression and displacement s	Yes	Not tested	Moderate	Yes	Only suitable for housed animals
Hoof overgrowth	Moderat e	Yes	Not specific	Yes	Only suitable for housed animals
Integument alterations	Yes	Yes	Good	Yes	
Lameness	Yes	Yes	Good	Yes	
Faecal soiling of breech	Yes	Yes	Moderate	Yes	
Faecal egg count	Yes	Yes	Good	No	
Wool/skin condition	Yes	Yes	Moderate	Yes	
Mucosa colour	Yes	Moderate	Good	Yes	
Eye condition	Yes	Not tested	Good	Yes	
Eye discharge	Yes	Not tested	Good	Yes	
Hampered respiration	Yes	Not tested	Good	Yes	
Coughing	Yes	Not tested	Good	Yes	
Nasal discharge	Yes	Not tested	Good	Yes	
Swollen joints/callus	Yes	Poor	Good	Yes	
Udder symmetry	No	Not tested	Poor	Yes	
Udder lesions	Yes	Not tested	Good	Yes	Generally low incidence
Udder temperature	Yes	Not tested	Moderate	Yes	
Presence of udder fibroids	Yes	Not tested	Good	Yes	Generally low incidence
Ear damage	Yes	Not tested	Good	Yes	Tagging is a legal requirements in many countries, only assess

					damage associated with poor practice.
Tail docking – absence of full tail	Yes	Not tested	Good	Yes	Tail docking legal in many countries, only assess compliance with the law.
Teeth grinding	Yes	Not tested	Good	No	Potentially feasible at group level only
Social withdrawal	Yes	Not tested	Not specific	Yes	
Facial expression	Yes	Yes	Good	Moderate	
Pain postures	Not tested in adult sheep	Not tested	Moderate	Yes	
Vocalisations	Yes	Not tested	Poor	Moderate	Feasible at group level only
Behavioural synchrony	Yes	Not tested	Poor	Yes	
Abnormal behaviour	Yes	Not tested	Good	Moderatel y	Generally very low incidence
Vigilance	Yes	Not tested	Poor	Yes	
Response to surprise	Yes	Not tested	Moderate	Moderatel y	
Novel object test	Yes	Not tested	Moderate	No	Between farm variation in feasibility
Human approach test	Yes	Not tested	Good	Yes	
Fear test	Yes	Repeatabilit y good, reliability not tested	Good	Yes	Housed animals only
Response to milking	Yes	Repeatabilit y good, reliability not tested	Moderate	Moderatel y	Dairy animals only
Qualitative Behavioural	Yes	Yes	Not specific	Yes	

Assessment (QBA)					
Play behaviour	Yes	Not tested	Good	No	Low play incidence in adult animals

References

Boissy, A., Dumont, B. (2002). Interactions between social and feeding motivations on the grazing behaviour of herbivores: sheep more easily split into subgroups with familiar peers. Appl. Anim. Behav. Sci. 79, 233–245.

Destrez, A., Deiss, V., Leterrier, C., Boivin, X., Boissy, A. 2013. Long-term exposure to unpredictable and uncontrollable aversive events alters fearfulness in sheep. Animal 7, 476-484.

Hutson, G.D. 1982. Flight distance in Merino sheep. Anim Prod 35, 231-235.

Lauber, M., Nash, J. a., Gatt, A., Hemsworth, P.H., 2012. Prevalence and Incidence of Abnormal Behaviours in Individually Housed Sheep. Animals 2, 27–37.

Lowe, T.E., Gregory, N.G., Fisher, A.D., Payne, S.R. 2002. The effects of temperature elevation and water deprivation on lamb physiology, welfare, and meat quality. Austr, J Agric Res 53, 707-714.

Pines, M.K., Petherick, J.C., Gaughan, J.B., Phillips, C.J.C., 2007. Stakeholders ' assessment of welfare indicators for sheep and cattle exported by sea from Australia. Anim. Welf. 16, 489–498.

Stott, A., Vosough Ahmadi, B., Dwyer, C., Kupiec, B., Morgan-Davies, C., Milne, C., Ringrose, S., Goddard, P., Phillips, K., Waterhouse, A., 2012. Interactions between profit and welfare on extensive sheep farms. Anim. Welf. 21, 57–64.

Tadich, N., Gallo, C., Brito, M.L., Broom, D.M. 2009. Effects of weaning and 48 h transport by road and ferry on some blood indicators of welfare in lambs. Livest Sci 121, 132-136.

Veksler, H.J., Schuh, A., Coppola, M., Decaminada, E., Miralles, M., Ghirardi, M., 2008. Effects of husbandry practices and animal welfare on reproductive indicators in sheep. Reprod Dom Anim. 43, 209.

Wemelsfelder, F., Farish, M., 2004. Qualitative categories for the interpretation of sheep welfare: a review. Anim. Welf. 13, 261–268.
